# Developmental Inhibition of Long Intergenic Non-Coding RNA, *HOTAIRM1*, Impairs Dopamine Neuron Differentiation and Maturation

**DOI:** 10.3390/ijms22147268

**Published:** 2021-07-06

**Authors:** Xiaoying Cui, Renata Ap. Nedel Pertile, Zilong Du, Wei Wei, Zichun Sun, Darryl W. Eyles, James P. Kesby

**Affiliations:** 1 Queensland Centre for Mental Health Research, Wacol, QLD 4076, Australia; x.cui@uq.edu.au (X.C.); d.eyles@uq.edu.au (D.W.E.); 2 Queensland Brain Institute, University of Queensland, Brisbane, QLD 4072, Australia; r.pertile@uq.edu.au (R.A.N.P.); zilong.du@uq.edu.au (Z.D.); royweiwei12@foxmail.com (W.W.); zichun.sun@uq.net.au (Z.S.); 3 QIMR Berghofer Medical Research Institute, Herston, QLD 4029, Australia

**Keywords:** in utero electroporation, lmx1a, Nurr1, tyrosine hydroxylase, mesencephalon

## Abstract

The dopaminergic (DA) system is important for a range of brain functions and subcortical DA development precedes many cortical maturational processes. The dysfunction of DA systems has been associated with neuropsychiatric disorders such as schizophrenia, depression, and addiction. DA neuron cell fate is controlled by a complex web of transcriptional factors that dictate DA neuron specification, differentiation, and maturation. A growing body of evidence suggests that these transcriptional factors are under the regulation of newly discovered non-coding RNAs. However, with regard to DA neuron development, little is known of the roles of non-coding RNAs. The long non-coding RNA (lncRNA) HOX-antisense intergenic RNA myeloid 1 (*HOTAIRM1*) is present in adult DA neurons, suggesting it may have a modulatory role in DA systems. Moreover, *HOTAIRM1* is involved in the neuronal differentiation in human stem cells suggesting it may also play a role in early DA neuron development. To determine its role in early DA neuron development, we knocked down *HOTAIRM1* using RNAi in vitro in a human neuroblastoma cell line, and in vivo in mouse DA progenitors using a novel in utero electroporation technique. *HOTAIRM1* inhibition decreased the expression of a range of key DA neuron specification factors and impaired DA neuron differentiation and maturation. These results provide evidence of a functional role for *HOTAIRM1* in DA neuron development and differentiation. Understanding of the role of lncRNAs in the development of DA systems may have broader implications for brain development and neurodevelopmental disorders such as schizophrenia.

## 1. Introduction

The dopaminergic (DA) system plays a central role in key brain functions including voluntary movement, cognition, motivation, and reward behaviour [1,2,3]. Alterations in the early development of DA neurons by both genetic [4] and environmental factors [5,6] can adversely affect how DA circuits are formed. Therefore, early changes in DA development may be an antecedent pathology in neurodevelopmental disorders that also feature dopaminergic dysfunction, such as schizophrenia [7]. Understanding the molecular mechanisms that regulate the maturation of the DA system will lay the groundwork for understanding how brain development shapes adult brain function and contributes to these disorders.

In both human and mouse embryos, almost all DA neurons are born early in gestation following similar temporal/spatial expressions of many DA transcription factors [8]. In a mouse, the majority of DA neurons are born on embryonic day (E) 11, preceding the appearance of tyrosine hydroxylase (TH; the rate-limiting enzyme in DA synthesis) [9,10]. Innervation of the striatum and cortex by DA neurons begins at E14 and E15, respectively, and functional striatal DA release is evident by E18 [11,12]. The phenotypic diversity, position, and projection sites of DA neurons are organised by a complex web of signalling cascades, which are tightly regulated by a range of specification and transcription factors as DA neurons differentiate and mature [4,13,14,15,16]. These factors play crucial and diverse roles in generating specific subsets of DA neurons.

Non-coding RNAs are emerging as factors that can regulate multiple signalling cascades via epigenetic mechanisms, translation regulation, and post-translational modification [17,18,19]. Non-coding RNAs are regulatory RNAs that are not translated into proteins. On the basis of the number of the nucleotides, non-coding RNAs are classified into short (<200 bp) and long non-coding RNAs (>200 bp; lncRNA). LncRNAs regulate brain development, maintenance, and function, taking part in fundamental processes such as neurogenesis [20], synapse formation [21], and neuronal subtype specification [22]. Recent research has also established that multiple non-coding RNAs are present in DA neurons [23]. However, the role of lncRNAs in DA neuron development is not well understood.

LncRNAs interfere with gene transcription and protein expression through mechanisms across multiple layers including altering chromatin structures, epigenetic regulation, and pre- and post-transcriptional modulation. For example, nuclear lncRNAs interact with DNA by sequence complementarity to modulate gene expression and maintain pluripotency in embryonic stem cells [24]. LncRNAs also act as a transcriptional enhancer (if localised at the promoter region) to promote gene expression [25]. Post-transcriptionally, lncRNAs containing binding sequences for microRNAs can act as a microRNA sponge to block microRNA-induced target mRNA destabilisation [26,27]. Recently some lncRNAs were found to be able to encode small proteins or polypeptides [28].

One particular lncRNA, the long intergenic non-coding RNA HOX-antisense intergenic RNA myeloid 1 (*HOTAIRM1*), is present in adult human DA neurons and may regulate key dopaminergic factors. For example, studies on the post-mortem brain of individuals with cocaine addictions have found that *HOTAIRM1* levels were correlated with the expression of genes coding for DA neuronal markers, including TH, the DA transporter (DAT), and the nuclear receptor related 1 protein (Nurr1) [29]. *HOTAIRM1* levels increase 50-fold when human induced pluripotent stem (iPS) cells are differentiated into neurons [30], indicating the potential involvement of *HOTAIRM1* in neuronal fate determination. *HOTAIRM1* is also a key factor for retinoic acid (RA) signalling within hematopoietic stem cells [31]. In the developing mesencephalon, RA induces the terminal differentiation of DA neurons within the substantia nigra [32], providing a mechanism for *HOTAIRM1* to influence DA neuron development. Taken together, these results suggest *HOTAIRM1* may be an important factor in the differentiation and maturation of DA neurons.

In this study, we specifically investigated the function of *HOTAIRM1* in DA neuron differentiation and maturation. Our studies which inhibited *HOTAIRM1* in human cell lines provide evidence of a role for *HOTAIRM1* in DA neuronal differentiation. We also adapted a triple-electrode in utero electroporation protocol [33] to transfect small interfering RNA (siRNA) against *HOTAIRM1* into mesencephalic DA progenitors in mouse embryos (E11). We then determined the downstream in vivo effects on target factors in DA development. Our studies highlight a potential role for lncRNAs, including *HOTAIRM1*, as modulatory factors in early dopamine neuron development.

## 2. Results

### 2.1. Decreasing HOTAIRM1 Reduced the Expression of DA Neuron Markers in SHSY5Y Cells

**Figure 1:**
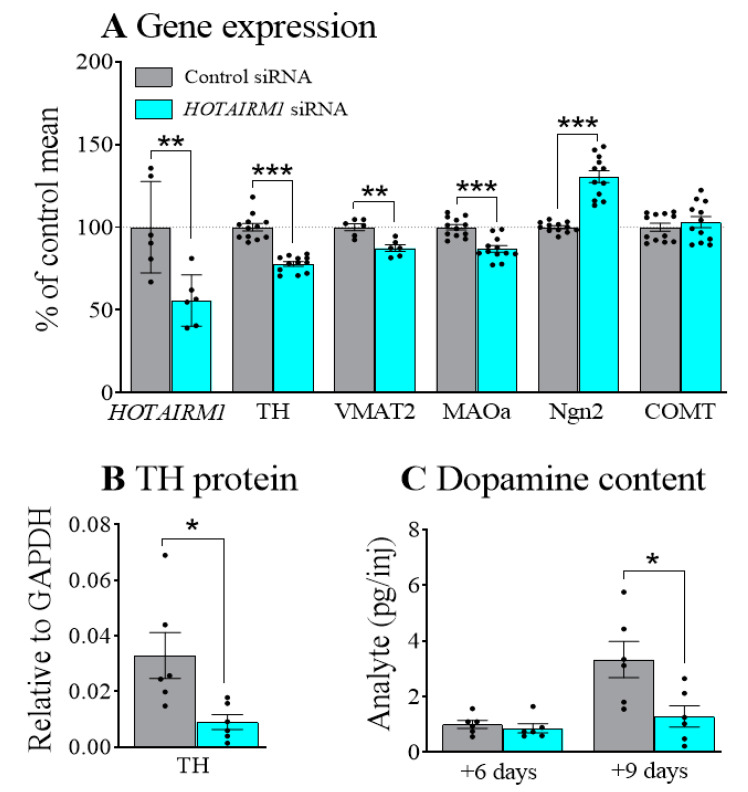
Effects of *HOTAIRM1* attenuation on SHSY5Y cells. SHSY5Y cells were transfected with siRNA against *HOTAIRM1* for 24 h followed by differentiation in the presence of retinoic acid. Inhibition of *HOTAIRM1* reduced mRNA levels of TH, VMAT2, and MAOa, but increased Ngn2 after 48 h of differentiation relative to control siRNA (**A**). Changes in the protein levels of TH after *HOTAIRM1* attenuation (**B**). Support for the observed changes in TH gene expression. Decreased dopamine content after *HOTAIRM1* attenuation was also observed after 9 days of differentiation (**C**). *siRNA*, small interfering RNA; *HOTAIRM1*, HOX-antisense intergenic RNA myeloid 1; *COMT*, Catechol-O-Methyltransferase; *TH*, tyrosine hydroxylase; *MAOa*, monoamine oxidase A; *Ngn2*, neurogenin 2; *VMAT2*, vesicular monoamine transporter 2. Data are expressed as Mean ± SEM. * *p* < 0.05, ** *p* < 0.01, *** *p* < 0.001.

After confirming the expression of *HOTAIRM1* in SHSY5Y cells and siRNA efficiency (Figure S1), we used RA to induce the differentiation of cells into DA neurons. We first knocked down the levels of *HOTAIRM1* in SHSY5Y cells using siRNA against *HOTAIRM1* for 24 h and then differentiated the cells for 48 h in the presence of RA (Figure 1A). We examined the levels of early DA neuronal specification markers including the LIM homeodomain gene (lmx1a) and Nurr1, and DA neuronal markers such as TH and DAT. We also assessed the factors that are involved in DA metabolism, e.g., vesicular monoamine transporter (VMAT2) and an enzyme responsible for the intraneural metabolism of DA, monoamine oxidase (MAOa). Aldehyde dehydrogenase-1a1 (AHD2), an enzyme only expressed in DA neurons within substantia nigra [13] was also examined. Our results showed that attenuating *HOTAIRM1* (*F*_1,10_ = 11.8, *p* < 0.01) reduced the expression of TH (*F*_1,22_ = 70.4, *p* < 0.001), VMAT2; (*F*_1,11_ = 18.7, *p* < 0.01), and MAOa; (*F*_1,22_ = 25.7, *p* < 0.001). In contrast, the proneural gene that modulates neuronal differentiation, neurogenin 2 (Ngn2), was increased after *HOTAIRM1* attenuation (*F*_1,22_ = 66.9, *p* < 0.001). Catechol-O-methyl transferase (COMT), a key enzyme in dopamine metabolism, but not expressed in DA neurons, was not affected by *HOTAIRM1* attenuation. Levels of lmx1a, Nurr1, DAT, and AHD2 were extremely low, but similar profiles were observed (Figure S2).

To confirm that changes in gene expression were complemented by changed protein levels, we quantified the level of the TH protein after 48 h of differentiation (Figure 1B and Figure S3). This confirmed that *HOTAIRM1* attenuation significantly decreased TH protein levels (*F*_1,10_ = 7.5, *p* < 0.05). As TH is the rate limiting enzyme in DA synthesis, we determined the level of DA in cultures after 48 h, and after 6 days and 9 days of differentiation. Levels were undetectable at 48 h and close to the limits of detection at 6 days. However, after 9 days of differentiation DA levels were significantly lower in cells previously transfected with siRNA against *HOTAIRM1* compared with the control siRNA (Figure 1C; *F*_1,10_ = 7.3, *p* < 0.05).

### 2.2. HOTAIRM1 Expression Increases during Early Development in the Ventral Midbrain

**Figure 2:**
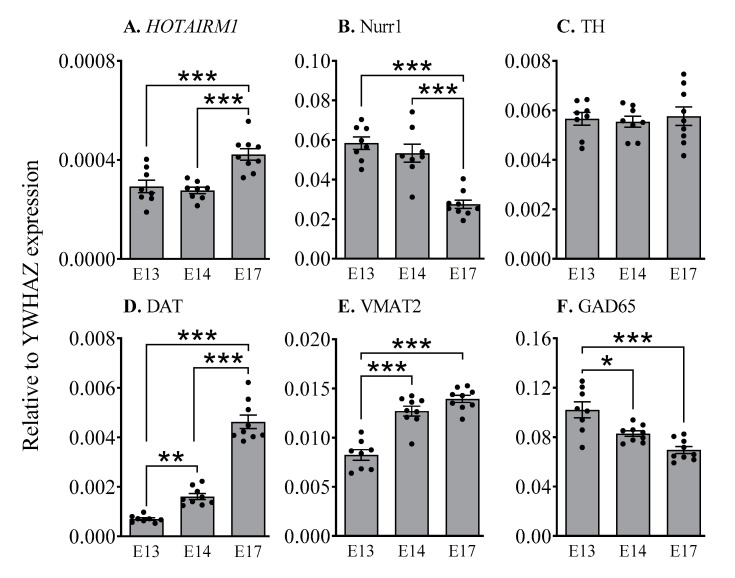
Temporal changes in *HOTAIRM1* expression and DA neuron maturation. Expression levels of *HOTAIRM1* (**A**) DA-related factors (**B**–**E**) and GABAergic marker, GAD65 (**F**) in the ventral midbrain of mice from E13 to E17. *HOTAIRM1*, HOX-antisense intergenic RNA myeloid 1; *Nurr1*, nuclear receptor related 1 protein; *TH*, tyrosine hydroxylase; *DAT*, dopamine transporter; *VMAT2*, vesicular monoamine transporter 2; *GAD65*, Glutamate decarboxylase 65. Data are expressed as Mean ± SEM. * *p* < 0.05, ** *p* < 0.01, *** *p* < 0.001.

To determine whether *HOTAIRM1* levels changed during the development period, we analysed the ventral midbrain of non-electroporated mice at E13, E14, and E17, which includes the primary period of early DA neuron maturation (Figure 2). YWHAZ (tyrosine 3-monooxygenase/tryptophan 5-monooxygenase activation protein, zeta polypeptid3) was used as a housekeeping gene, rather than the more common HPRT, since it is one of the most stable genes in the developing brain [34]. *HOTAIRM1* expression increased as demonstrated by a significant main effect of *Age* (*F*_2,22_ = 14.2, *p* < 0.001), and the levels of *HOTAIRM1* were significantly greater at E17 than the levels observed at E13 (*p* < 0.001) and E14 (*p* < 0.001). The significant main effects of *Age* were also observed for Nurr1 (*F*_2,22_ = 25.7, *p* < 0.001), DAT (*F*_2,23_ = 130.2, *p* < 0.001), VMAT2 (*F*_2,23_ = 39.9, *p* < 0.001), and GAD65 (*F*_2,23_ = 15.8, *p* < 0.01) across this period, but not for TH (statistical comparisons can be seen in Figure 2). These findings are supported by other studies showing that Nurr1 levels tend to peak between E13 and E15 [35] and are highest in immature DA neurons prior to TH expression [36]. This precedes and stimulates the expression of dopaminergic factors such as VMAT2 and DAT.

### 2.3. Electroporation of Mesencephalic Dopamine Progenitors in the Mouse 

**Figure 3:**
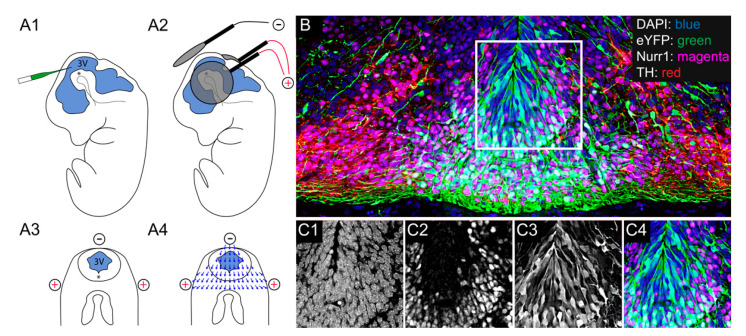
Electroporation of mesencephalic dopamine progenitors in the mouse. Plasmid and siRNA (control or active) solution were injected into the third ventricle (3V) of the embryonic day 11 mouse embryo (**A1**) in order to target the ventral mesencephalon (**A2**,**A3**). Two positive electrodes were placed laterally and ventrally to the mesencephalon and a single negative electrode was placed dorsal to the mesencephalon (**A2**,**A3**). This positioning allows for the generation of a ventral electrical field vector (**A4**). Example image of E13 ventral midbrain after electroporation at E11, with eYFP expression (green) identifying transfected cells (**B**). Separate DAPI (**C1**), Nurr1 (**C2**), eYFP (**C3**) and composite (**C4**) from inset in (**B**). *siRNA*, small interfering RNA; *DAPI*, 4′,6-diamidino-2-phenylindole; *eYFP*, enhanced yellow fluorescent protein; *Nurr1*, nuclear receptor related 1 protein; *TH*, tyrosine hydroxylase.

The plasmid and siRNA (control or active) solutions were injected into the third ventricle (3V) of an E11 mouse embryo and a triple-probed electroporation was applied. The co-transfection of plasmids encoding fluorescent proteins and siRNA is a common strategy for identifying transfected cells, with efficient co-transfection observed in most cells [37]. Two days later, we observed that the eYFP was successfully transfected into DA progenitors within the ventral midbrain (Figure 3 and Figure S4).

### 2.4. Inhibition of HOTAIRM1 alters Markers of DA Differentiation and Maturation in the Developing Mesencephalon

#### 2.4.1. E13, Two-Days after Transfection

**Figure 4:**
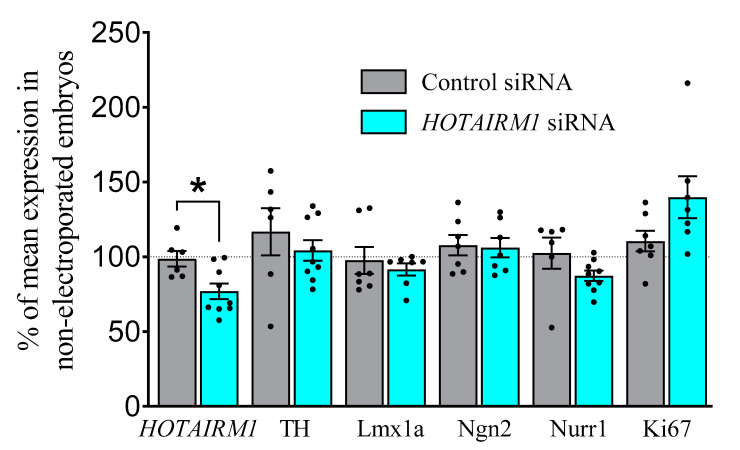
Effects of in vivo attenuation of *HOTAIRM1* on DA factors at E13. Expression levels of *HOTAIRM1*, TH, lmx1a, Ngn2, Nurr1, and Ki67 in the ventral midbrain at E13 after transfection with siRNA against *HOTAIRM1* at E11. Results of each embryo were normalised to the mean expression of non-electroporated embryos from the same litter. *HOTAIRM1* levels were significantly reduced after targeted siRNA transfection, but no effects on DA markers (TH, lmx1a, Ngn2, and Nurr1) or a marker of proliferation (Ki67) were observed. *siRNA*, small interfering RNA; *HOTAIRM1*, HOX-antisense intergenic RNA myeloid 1; *TH*, tyrosine hydroxylase; *Lmx1a*, LIM homeodomain gene 1a; *Ngn2*, neurogenin 2; *Nurr1*, nuclear receptor related 1 protein. Data are expressed as Mean ± SEM. * *p* < 0.05.

The electroporation of siRNA against *HOTAIRM1* into DA progenitors at E11 significantly reduced *HOTAIRM1* levels at E13 (*F*_1,13_ = 4.9, *p* < 0.05), as expected (Figure 4). However, this did not affect any early markers of DA differentiation or maturation such as TH, lmx1a, Ngn2, or Nurr1 (for raw data, see Supplementary Materials Table S3). Similarly, no change was observed for the proliferation marker, Ki67.

#### 2.4.2. E17, Six-Days after Transfection

**Figure 5:**
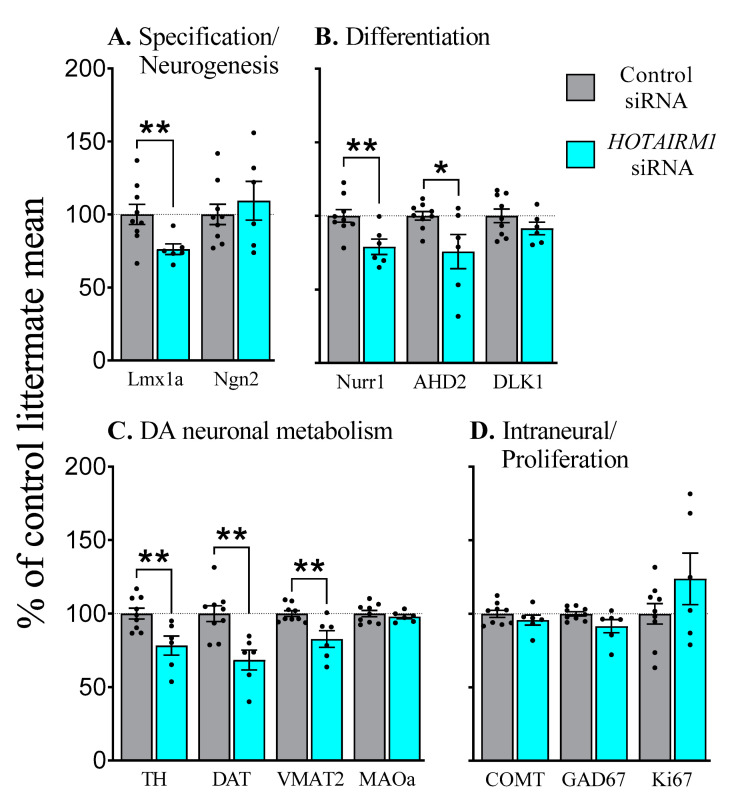
Effects of in vivo attenuation of *HOTAIRM1* on DA factors at E17. Expression levels in the ventral midbrain at E17 after transfection with siRNA against *HOTAIRM1* at E11. All litters included both control and *HOTAIRM1* siRNA transfected embryos. Therefore, results are presented as the percentage of the mean expression of embryos transfected with control siRNA within each litter. *HOTAIRM1* attenuation reduced the levels of factors involved in DA specification (**A**) differentiation (**B**) and DA neuronal metabolism (**C**). However, no effects were observed for factors expressed in non-DA cell types or proliferative markers (**D**). *siRNA*, small interfering RNA; *HOTAIRM1*, HOX-antisense intergenic RNA myeloid 1; *Lmx1a*, LIM homeodomain gene 1a; *Ngn2*, neurogenin 2; *Nurr1*, nuclear receptor related 1 protein; *DLK1*, Delta like non-canonical Notch Ligand 1; *AHD2*, aldehyde dehydrogenase-1a1; *TH*, tyrosine hydroxylase; *DAT*, dopamine transporter; *VMAT2*, vesicular monoamine transporter 2; *MAOa*, monoamine oxidase A; *COMT*, Catechol-O-Methyltransferase; *GAD65*, Glutamate decarboxylase 65. Data are expressed as Mean ± SEM. * *p* < 0.05, ** *p* < 0.01.

In contrast to our results at E13, a range of key factors were affected at E17 suggesting *HOTAIRM1* plays a specific role in the specification and differentiation of DA neurons (Figure 5; for raw data, see Supplementary Materials Table S4). Prior *HOTAIRM1* attenuation decreased the levels of lmx1a (*F*_1,13_ = 7.0, *p* < 0.05) but not Ngn2, highlighting a role in specification but not neurogenesis (Figure 5A). Furthermore, decreased levels of the differentiation factors Nurr1 (*F*_1,13_ = 9.5, *p* < 0.01) and AHD2 (*F*_1,13_ = 6.0, *p* < 0.05) were observed, but Delta like non-canonical Notch ligand 1 (DLK1) levels were similar to those in the controls (Figure 5B). Most of the enzymes expressed within DA neurons that are responsible for DA production and metabolism, including TH (*F*_1,13_ = 9.9, *p* < 0.01), DAT (*F*_1,13_ = 13.6, *p* < 0.01), and VMAT2 (*F*_1,13_ = 11.2, *p* < 0.01) were also decreased by *HOTAIRM1* attenuation (Figure 5C). No changes were observed in the levels of COMT, or Glutamate decarboxylase 65 (GAD65), a marker of GABAergic neurons (Figure 5D). Similarly, the proliferation marker Ki67 was also unchanged.

## 3. Discussion

The results of this study suggest that lncRNA *HOTAIRM1* is involved in early DA neuron development, with a role in both specification and differentiation. Reducing the levels of *HOTAIRM1* led to subsequent decreases in the levels of DA transcription factors that are important in neuronal specification (lmx1a) and differentiation (Nurr1 and AHD2), and delayed DA neuron maturation (TH, DAT, and VMAT2). These results were confirmed in both in vitro studies in a human cell line and in vivo studies in mouse embryos. These data highlight a role for *HOTAIRM1* in early DA development, which may have long-term consequences for adult brain function.

In this study, we show that low levels of *HOTAIRM1* are present in the developing mesencephalon of the mouse and increase during development alongside DA neuron maturation. The level of *HOTAIRM1* expression is low but this is a common feature of most of the non-coding RNAs [38,39,40,41]. *HOTAIRM1* is expressed in adult human DA neurons and its levels have been associated with the expression of DA neuron markers TH and DAT [29]. Here, we further revealed that *HOTAIRM1* is required for the expression of multiple established protein-coding transcriptional factors essential for generating a DA phenotype in the developing mouse brain. For example, lmx1a is considered the master regulator of the DA progenitor identity [14] and delineates the DA progenitor zone. Post-mitotic DA neurons begin to express Nurr1, which is critical for driving the expression of mature DA neuronal markers such as TH, DAT, and VMAT2, before decreasing in expression [36]. Alterations in any of these proteins, such as those observed after *HOTAIRM1* attenuation, can lead to persistent changes in DA function and connectivity.

*HOTAIRM1* is expressed at high abundance in the bone marrow and has been identified as a key mediator of RA-induced myeloid lineage differentiation in human hematopoietic cells [31]. Aprea and colleagues have shown that RA increased the level of *HOTAIRM1* when human iPS cells were differentiated into neurons and predicted that this lncRNA might be involved in neuron differentiation [42]. Our in vitro data suggest that decreasing *HOTAIRM1* could impair or delay DA neuron maturation, as the knockdown of this lncRNA reduced the expression of the mature DA neuron markers TH and VMAT2 but increased the expression of the immature DA marker Ngn2. This was also accompanied by decreases in the TH protein, and decreased levels of DA after longer periods of differentiation. Our in vivo data indicates that *HOTAIRM1* could be a mediator for RA-induced DA neuronal differentiation more specifically, as knockdown reduced the expression of specification and differentiation genes such as lmx1a and Nurr1. The mechanisms that explain how *HOTAIRM1* reduction diminishes DA neuronal differentiation are not clear; one plausible mechanism may be that *HOTAIRM1* serves as a molecular scaffold to recruit epigenetic modifiers on the promoters of RA-modulated genes such as Ngn2. Rea and colleagues have shown that *HOTAIRM1* epigenetically modulates Ngn2 expression via the polycomb repressive complex 2 (PRC2) to recruit the repressive marker trimethylated H3K27 on the promoter of Ngn2. Reducing *HOTAIRM1* led to a decreased H3K27me3 deposition on the promoter of Ngn2, which within 3 days of RA-induced differentiation resulted in an upregulation of Ngn2 mRNA expression in SHSY5Y cells [43]. Ngn2 is known to regulate DA neuron differentiation [44]. Our in vitro results showed that reducing *HOTAIRM1* enhanced Ngn2 expression; however, our in vivo study did not detect the alterations in Ngn2 by *HOTAIRM1* reduction. This could be due to the broader expression of Ngn2 within the ventricular zone, which extends laterally to the DA progenitor domain [45]. As our triple-probe electroporation specifically transfected *HOTAIRM1* siRNAs into the DA progenitors, it likely had a minimal effect on neuronal cells lateral to the DA progenitors. Therefore, a real time PCR will not detect the effect of reducing *HOTAIRM1* on overall Ngn2 expression within the ventral midbrain. To elucidate the epigenetic regulation of *HOTAIRM1* on Ngn2 expression, sorting siRNA transfected DA progenitors will be required in future studies.

During DA neuron differentiation in the mouse mesencephalon, the transcriptional factor Nurr1 and the paired-like homeodomain transcription factor 3 (Pitx3) induced the expression of AHD2. AHD2 is important in the synthesis of RA, which promotes DA neuron maturation [32,46]. *HOTAIRM1*-mediated RA signalling is one potential mechanism that explains how *HOTAIRM1* regulates DA neuron differentiation in the mouse mesencephalon. Our in vivo data support this hypothesis, with AHD2 levels reduced at E17 after prior *HOTAIRM1* attenuation. AHD2 expression is located primarily in DA neurons within the substantia nigra [13]. Thus, these data highlight that *HOTAIRM1* may play a larger role in the differentiation of DA neurons that subsequently populate the substantia nigra, as compared with those that populate the ventral tegmental area. Whether these changes impact the phenotypic development of the midbrain in vivo remains unknown. We have shown that the mRNA expression of key factors in DA neuron development are altered, and in vitro this translates to altered levels of TH protein as well as DA neurotransmitter content. Future studies following the maturation of DA neurons and persistent outcomes into adulthood are required to demonstrate whether this is the case.

Although little is known regarding the role of *HOTAIRM1* in the brain, its role in regulating proliferation and differentiation has been extensively studied in cancer cells. However, whether *HOTAIRM1* regulates proliferation remains controversial [31,47,48]. Our results show no indication for a role of *HOTAIRM1* in the proliferation of DA neuron progenitors or more generally in the ventral mesencephalon. For example, levels of ki67, which represent general cellular proliferation, were not altered at any time point and, if anything, appeared to be mildly increased. The lack of alterations in Ngn2 and DLK1, key factors in DA neurogenesis and differentiation, further support the argument that reduced *HOTAIRM1* did not have a generalised effect on the proliferation of DA progenitors. Similarly, we observed no evidence that *HOTAIRM1* attenuation altered the proliferation of other ventral midbrain cell types. Expression of GAD65, a marker of GABAergic cell types, was not changed relative to the control siRNA.

One limitation of the present study is that the effect of *HOTAIRM1* on HOXa genes was not explored. As intergenic non-coding RNAs, such as other non-coding RNAs in *HOTAIR* family, *HOTAIRM1* is well known to modulate HOXa gene clusters in a cis action [31,49,50]. However, because the levels of HOXa genes are extremely low in SHSY5Y cells and the embryonic ventral mesencephalon, this canonical pathway could not be examined. Moreover, the in vivo changes in DA transcription factors were observed across the whole ventral midbrain, even though only ~8% of cells were routinely transfected with siRNA. Future studies are required to assess whether non-autonomous cellular mechanisms are impacting the expression profiles in neighbouring cells.

In conclusion, our in vitro and in vivo data have consistently shown that the knockdown of *HOTAIRM1* attenuated RA-mediated DA neuron differentiation, suggesting a role for this lncRNA in DA neuron development. These novel data highlight that *HOTAIRM1*, even when expressed at low abundance in DA neurons, plays an important regulatory role in neuronal specification and maturation.

## 4. Materials and Methods

### 4.1. Animals

Time-mated female CD1 mice were purchased (ARC, Perth, WA, Australia) and housed in groups of 2–4 (until after surgery) in ventilated OptiMice cages (Animal Care Systems, Centennial, CO, USA) at 21 °C, 40–60% humidity, and with a 12 h light and dark cycle (lights on 07:00 h) with *ad libitum* access to food and water. All animal procedures were approved by The University of Queensland Animal Ethics Committee.

### 4.2. Cell Culture and siRNA Transfection

Human SHSY5Y was purchased from American Type Cell Collection (ATCC). Cells were grown in the media containing DMEM/F12 (Thermo Fisher Scientific Australia Pty Ltd., Scoresby, VIC, Australia), a 10% fetal calf serum (FCS, Hyclone, GE Healthcare Australia Pty Ltd., Parramatta, NSW, Australia), a GlutaMAX supplement (Thermo Fisher Scientific), 100 U/mL penicillin and 100 µg/mL streptomycin (Thermo Fisher Scientific). SHSY5Y cells were seeded at a concentration of 100,000 cell/mL into each well of a 24 well plate. On the second day, the growth medium was changed to an Opti-MEM serum free media (Thermo Fisher Scientific). siRNAs (20nM; Thermo Fisher Scientific) against *HOTAIRM1* (human *HOTAIRM1* siRNA [n507510]) or scrambled control (Negative control #1 4457289) were mixed with Lipofectamine^TM^ RNAiMAX transfection reagent and added into the serum free media. A total of 24 h after siRNA transfection, the serum free media was replaced with differentiation media containing DMEM/F12, 1%FCS and all-trans retinoic acid (RA, 10 µM, Sigma-Aldrich, St. Louis, MO, USA). For RNA extraction, SHSY5Y cells were collected into a lysis buffer RLT (Qiagen Chadstone, VIC, Australia). For protein quantification, cells were collected into a lysis buffer (150 mM NaCl, 20 mM HEPES, 2mM EDTA, 0.1% Triton100, pH 7.4) containing a protease inhibitor cocktail (Roche Diagnostics GmbH, Mannheim, Germany).

### 4.3. Western Blot

Protein concentrations were examined using a Piece BCA protein assay kit (Thermo Fisher Scientific). A total of 10µg of the protein of each sample was loaded in a lane of the precast NUPAGE 4–12% Bis-Tris gels (Thermo Fisher Scientific). Proteins were then transferred to a polyvinylidene fluoride (PVDF) membrane for two hours at 400 mA. The membrane was blocked for 30 min using an Intercept blocking buffer (LI-COR) and the primary antibodies against TH (1:2500, Abcam, Cambridge, UK) or GAPDH (1:10,000, Millipore, Burlington, MA, USA) were applied and incubated overnight at 4 °C. After being washed with 1x PBST (0.1% Tween 20 in PBS) 4 times, the membrane was incubated with the fluorescent conjugated secondary antibodies (IRDye680RD anti-mouse antibody [1:20,000] or IRDye800CW anti-rabbit antibody [1:30,000], LI-COR, Lincoln, NE, USA) for one hour at room temperature. Membranes were scanned using an Odyssey Fc Imaging system (Millennium Science, Mulgrave, VIC, Australia). The intensity of specific bands for TH or GAPDH and their relative expression were quantified using Odyssey software.

### 4.4. High Performance Liquid Chromatography

The in vitro cellular content of DA, its metabolite dihydroxyphenylacetic acid (DOPAC), and the major metabolite of serotonin, 5-hydroxy-indoleacetic acid (5-HIAA), were assessed after *HOTAIRM1* attenuation. Samples included 2 days (48 h), 6 days, and 9 days after siRNA transfection (in differentiation media). Dopaminergic metabolites (homovanillic acid and 3-Methoxytyromine) and serotonin were below detection levels are therefore were not reported. Analyte levels were measured by high performance liquid chromatography with electrochemical detection [51,52]. Each well of cells were collected in 0.2 mL of 0.1 M perchloric acid with 50 ng/mL deoxyepinephrine (internal standard). They were then homogenised on ice using probe sonication (2 × 5s at 60% amplitude; Vibra-Cell, Sonics & Materials, CT, USA) and centrifuged at 13,000 rpm for 5 min at 4 °C with the supernatant filtered by a 4 mm 0.22 μM nylon syringe filter (PM Separations, Capalaba, QLD, Australia). One 15 μL aliquot of sample supernatant was injected into the HPLC system, which consisted of a degasser, an autosampler with a thermostat and a binary HPLC pump (Model 1200, Agilent Technologies, Santa Clara, CA, USA), a Sunfire C18 column, 3.0 mm × 100 mm, 3.5 μm; (Waters Corporation, Taunton, MA, USA), and a Coulochem III (ESA Laboratories, Chelmsford, MA, USA) electrochemical detector. The mobile phase consisted of an 11.6% acetonitrile/50 mM citric acid and a 25 mM potassium dihydrogen phosphate buffer containing 1 mM of EDTA and 1.4 mM of octane sulfonic acid adjusted to a pH of 4.15 with sodium hydroxide. The flow rate was 0.6 mL/min. Detector settings were as follows: conditioning cell (Model 5020, ESA Laboratories) at +350 mV; analytical cell (Model 5011A, Thermo Fisher Scientific, Australia) with the first and second electrodes maintained at −150 and +250 mV, respectively. Data were quantified by calculating peak-area ratios for each specific analyte relative to the internal standard and calibrated using standard curves. Data were stored and processed with Chemstation software (Rev C.01.05, Agilent Technologies). Samples were corrected for dilution and expressed as picogram per injection. DA levels were not detectable at the 2-day time point and were close to the limit of detection at the 6-day time point.

### 4.5. In Utero Electroporation

Dams underwent abdominal surgery (1–2 cm) at E11.5 to expose the uterine horns. Using a pulled glass pipette attached to a picospritzer (Parker Hannifin, Cleveland, OH, USA), a solution including plasmid DNA encoding yellow fluorescent protein (pCAG-eYFP, 1.5 µg) and siRNA (3 ng/µL; Negative control #1 4457289 or *HOTAIRM1* [n520253], Thermo Fisher Scientific) was injected through the uterine wall into the third ventricle (3V) of mouse embryos. Electroporation of the ventral mesencephalon was accomplished using a triple electrode configuration (Figure 3) [33]. Two positive electrodes were placed laterally and ventrally to the mesencephalon. A single negative electrode was placed dorsal to the mesencephalon to create a ventral electrical field vector. Five electrical pulses were administered (amplitude, 35 V; duration, 50 ms; intervals, 950 ms) with a CUY21 SC electroporation system (Nepa Gene Co. Ltd., Chiba, Japan). The uterine horns were placed back into the abdominal cavity, and the dam was sutured and allowed to recover. For survival rates and transfection data see Supplementary Materials Table S1. This protocol transfected approximately 8% of all cells in the intermediate and mantle zones at E13, the majority of which were immature DA cells (see Figure S4).

### 4.6. Tissue Collection

For embryo collection, dams were euthanised via cervical dislocation and the uterine horns were removed and placed in ice-cold PBS. The ventral midbrain was rapidly dissected from each embryo and frozen in liquid nitrogen for subsequent RNA extraction and PCR.

### 4.7. RNA Extraction and Real Time PCR

Total RNA was extracted using a RNeasy Mini Kit (Qiagen) according to the manufacture’s protocol. A total of 1 µg RNA was used for cDNA synthesis which was conducted using a SensiFAST^TM^ cDNA synthesis Kit (Bioline Aust Pty Ltd., Eveleigh, NSW, Australia). The relative expression of genes of interest were measured using SensiFAST^TM^ SYBR^®^ No-ROX kit in a 384-well format in a Roche LightCycler480 system (Roche Life Science). Gene expression was established relative to housekeeping genes; HPRT (hypoxanthine-guanine phosphoribosyltransferase) was used for studies comparing active and control siRNAs. YWHAZ was used as a house keeping gene for studies looking at the temporal expression during development since it is one of the most stable genes in the developing brain [34]. The reaction conditions were as follows: 95 °C for 5 min, then 40 cycles of 95 °C for 10s, 60 °C for 10s, and 72 °C for 20s. For primer details see Supplementary Materials Table S2.

### 4.8. Statistics

All analyses were performed with IBM SPSS Statistics 26 (Armonk, NY, USA). Data were analysed using the multivariate analysis of variance (ANOVA), with *HOTAIRM1* and/or *Age* (for time-based expression profiles) as the between-subject factors. Data not meeting the assumption of homogeneity of variance were analysed using Greenhouse-Geiser adjusted degrees of freedom. When appropriate, post hoc comparisons were performed using Bonferroni analyses. Results were expressed as mean ± SEM. Differences were considered statistically significant at *p* < 0.05.

## Data Availability

Data is contained within the article or supplementary material.

## Supplementary Materials

The following are available online at https://www.mdpi.com/article/10.3390/ijms22147268/s1.
