# Supplementary material for: Developmental Inhibition of Long Intergenic Non-Coding RNA, HOTAIRM1, Impairs Dopamine Neuron Differentiation and Maturation
Source: Int J Mol Sci. 2021 Jul 6;22(14):7268. doi: 10.3390/ijms22147268 (PMC8306845; doi:10.3390/ijms22147268)
Supplement: Supplementary file 1 [file ijms-22-07268-s001.zip › ijms-1274961-supplementary.pdf]

Developmental inhibition of long intergenic non-coding RNA, *HOTAIRM1*, impairs dopamine neuron differentiation and maturation.

Xiaoying Cui[1,2], Renata Ap. Nedel Pertile[1], Zilong Du[1], Wei Wei[1], ZiChun Sun[1], Darryl W. Eyles[1,2], James P. Kesby[1,3]*

[1] Queensland Brain Institute, University of Queensland, Brisbane, Qld 4072, Australia

[2] Queensland Centre for Mental Health Research, Wacol, Qld 4076, Australia

[3] QIMR Berghofer Medical Research Institute, Herston, Qld 4029, Australia

* Corresponding author:

Dr. James Kesby

Queensland Brain Institute, The University of Queensland, Brisbane, QLD 4072, Australia.

Phone: +61 7 3346 6363

Fax: +61 7 3346 6301

Email: j.kesby@uq.edu.au

ORCID: 0000-0002-5814-8062

Key words: in utero electroporation, lmx1a, Nurr1, tyrosine hydroxylase, mesencephalon

SUPPLEMENTARY METHODS

Immunohistochemistry

Three dams underwent electroporation at E11 as described in the manuscript methods. At E13, 7 fetal brains were immediately dissected and immersion fixed in 4% paraformaldehyde overnight at 4 °C. Fixed brains were soaked in 30% sucrose solution prior to being embedded in Cryo-Embedding Compound (TissueTek, Emgrid Australia). Brain specimens were placed on a cryostat, and 20-μm coronal sections were cut through the entire mesencephalic region. One of every five sections were processed for immunohistochemistry. All sections were processed at the same time to exclude batch variation. The blocking and antibody buffer consisted of a phosphate-buffered saline solution with 0.5% Bovine serum albumin, 0.05% sapponin, 0.1% Triton X-100 (Sigma-Aldrich, USA) and 0.05% sodium azide. Primary antibodies used for immunostaining were chicken anti-GFP (1:750, ab13970, Abcam, USA), sheep anti-TH (1:200, NB300, NOVUS biologicals, USA) and rabbit anti-Nurr1 (1:500, sc-990, Santa Cruz Biotechnology, USA). Fluorophore-conjugated secondary antibodies were donkey anti-chicken conjugated Alexa-488 (1:1000, CF488A, Biotum, USA), donkey anti-sheep conjugated Alexa-555 (1:1000, A-21436, Thermo Fisher Scientific, Australia) and donkey anti-rabbit conjugated Alexa-647 (1:1000, A-31573 Thermo Fisher Scientific, Australia). Nuclei were stained using 4′,6-diamidino-2-phenylindole (DAPI, 1:1000, Sigma-Aldrich, Australia).

Image acquisition and analysis

All images of immunostained brain sections were immediately visualised and imaged using a Nikon Plan Apo Lambda 60x/1.4 NA oil-immersion objective on a spinning disk confocal microscope (Diskovery; Andor Technology, UK) built around a Nikon Ti-E body (Nikon Corporation, Japan) and equipped with two Zyla 4.2 sCMOS cameras (Andor Technology) and controlled by Nikon NIS software. Images were captured with a ROI of 1500 x 1500

pixels and at a z-step size of 0.3 µm, providing a voxel size 0.093 × 0.093 × 0.3 µm3. All images were deconvolved with Huygens Professional version 18.04 (Scientific Volume Imaging, The Netherlands). A single 2D slice from the middle of the z-stack was used for image analysis and quantification in CellProfiler 3 [www.cellprofiler.org; 1]. All sections where eYFP was visible in the ventral midbrain were imaged and analysed (at least 2 per embryo). Positive eYFP counts from these sections were then adjusted to reflect the total proportion of cells in the ventral midbrain (rather than the specific sections imaged, as there were no eYFP+ cells observed on the remaining sections).

Table S1. Electroporation survival and numbers

Electroporation age	Embryos per litter (# dams)	Electroporated embryos per litter	Surviving embryos (%)
E11.5	13.7 ± 0.8 (10)	6.5 ± 0.3	64.5 ± 8.6

Table S2. Real Time-PCR primers

Gene	Species	Accession No.	**Sequence (5'-3')**<br>Forward (F) and Reverse (R)
**HPRT**	Mus musculus	NM_013556	F: CAGTACAGCCCCAAAATGGT<br>R: TTGCGCTCATCTTAGGCTTT
HOTAIRM1	Mus musculus	NR131181	F: GAGTCGAGACTGCCTTCTGC<br>R: ACCCCCATTTTCAGTGTGGT
**TH**	Mus musculus	NM_009377	F: CGCCGTCCAATGAACCTT<br>R: CACTATGCCCACCCCCAG
**Lmx1a**	Mus musculus	NM_033652	F: AACCAGCGAGCCAAGATGAA<br>R:TGGGTGTTCTGTTGGTCCTGT
**Nurr1**	Mus musculus	NM_013613	F: GTGTTCAGGCGCAGTATGG<br>R: TGGCAGTAATTTCAGTGTTGGT
**NgN2**	Mus musculus	NM_009718	F:ATCTGGAGCCGCGTAGGAT<br>R:CATCAGTACCTCCTCTTCCTCCTT
**DAT**	Mus musculus	NM_010020	F: CATTGCCACATCCTCCATGG<br>R: CATTGCCACATCCTCCATGG
**COMT**	Mus musculus	NM_007744	F: GTCATCCTGATGGCCTCACT<br>R: TACCGTCTGAGTCGTTGCTG
**DLK1**	Mus musculus	NM_010052	F: GGACAGGCCATCTGCTTCAC<br>R: GCTCCTCGCCGCTGTTATAC
**AHD2**	Mus musculus	NM_001361504	F: GGAATACCGTGGTTGTCAAGCC<br>R: CCAGGGACAATGTTTACCACGC
**Ki67**	Mus musculus	NM_001081117	F: ATCATTGACCGCTCCTTTAGGT<br>R: GCTCGCCTTGATGGTTCCT
**MAOa**	Mus musculus	NM_173740	F: GGGGGCTGTCATCAAGTGCAT<br>R: TGGCAGGCATTGACCCATCTG
**VMAT2**	Mus musculus	NM_172523	F: GCAGTTGTGGTCCATGAG<br>R: GCAGTTGTGGTCCATGAG

**GAD65**	Mus musculus	NM_008078	F: AATGGTGTTTGATGGGAAGC<br>R: AGGGTTTGAGATGACCATGC
**YWHAZ**	Mus musculus	NM_011740	F: TAGGTCATCGTGGAGGGTCG<br>R: GAAGCATTGGGGATCAAGAACTT
**TH**	Homo sapiens	NM_199293	F: GCAGTTCTCGCAGGACATTG<br>R: TGGATGCGTGAGGCATAGC
**HPRT**	Homo sapiens	NM_000194	F: TGACCAGTCAACAGGGGACA<br>R: GCGACCTTGACCATCTTTGG
**MAOa**	Homo sapiens	NM_000240	F: GTGTCAGCCAAAGCATGGAG<br>R: GCAGCAGATAGTCCTGAAATGC
**VMAT2**	Homo sapiens	NM_003054	F: CCGTACATCCTCATTGCTGC<br>R: CCACCTCCCCATTTTGTGTG
**NgN2**	Homo sapiens	NM_024019	F:GCAAGCGTGGAAATTTAGGC<br>R:GCAATCCTCCCTCCTGATTT
HOTAIRM1	Homo sapiens	NR_038366	F:TAGTTATTGACCTGGAGACTGGTAGC<br>R:TCAGTGCACAGGTTCAAGCC

All Primers were purchased from Sigma-Aldrich.

SUPPLEMENTARY RESULTS

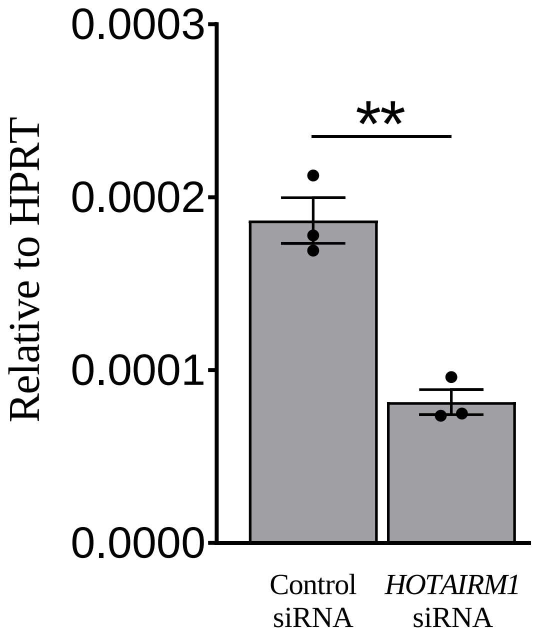

Figure S1. Expression levels of *HOTAIRM1* in SH-SY5Y cells after 24 h of transfection with 20 nM of control siRNA or *HOTAIRM1* siRNA. *HOTAIRM1* siRNA reduced *HOTAIRM1* levels by over 50% relative to control siRNA ($t_{1,4}$=6.9, p<0.01).

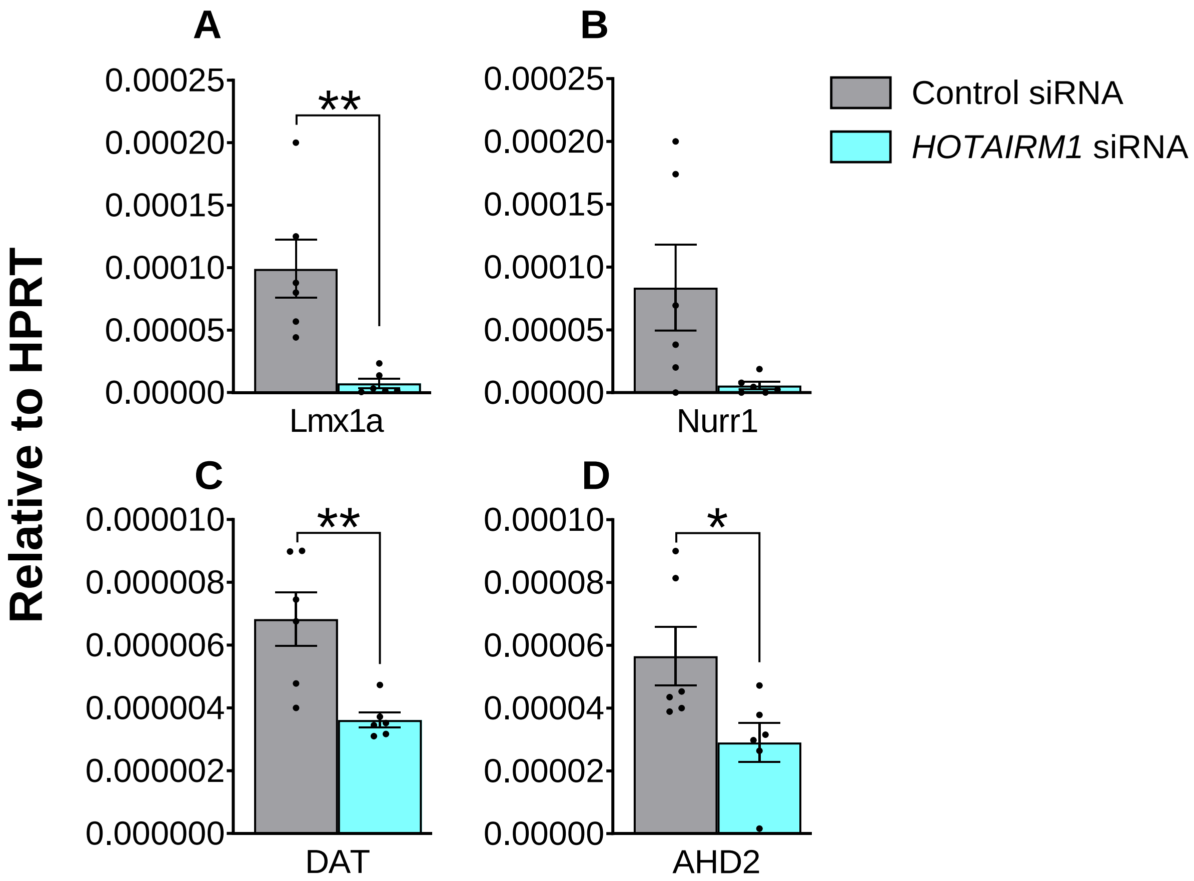

Figure S2. *In vitro* expression levels of Lmx1a (A), Nurr1 (B), DAT (C) and AHD2 (D) after *HOTAIRM1* siRNA transfection and 48 h differentiation in SHSY5Y cells. Levels were extremely low for all targets (requiring over 35 cycles of PCR). However, *HOTAIRM1* attenuation did significantly decrease Lmx1a ($F_{1,10}$=14.6, p<0.01), DAT ($F_{1,10}$=4.9, p<0.01) and AHD2 ($F_{1,10}$=6.3, p<0.05) levels. *siRNA*, small interfering RNA; *HOTAIRM1*, HOX-antisense intergenic RNA myeloid 1; *Lmx1a*, LIM homeodomain gene 1a; *Nurr1*, nuclear receptor related 1 protein; *DAT*, dopamine transporter; *AHD2*, aldehyde dehydrogenase-1a1. Data are expressed as Mean ± SEM. * p < 0.05, ** p < 0.01.

Table S3. In vivo E13 raw PCR data (^dCT)

	**Wild-type (n=7-8)**		**Control siRNA (n=6-7)**		***HOTAIRM1* siRNA (n=7-9)**	
	Mean	SEM	Mean	SEM	Mean	SEM
HOTAIRM1	0.009038	0.001658	0.007765	0.000489	0.006300	0.000602
TH	0.170731	0.021572	0.179574	0.024847	0.179280	0.012391
Lmx1a	0.218766	0.015728	0.199240	0.019078	0.216141	0.007426
Ngn2	0.068547	0.002837	0.072080	0.004636	0.073056	0.004613
Nurr1	1.995351	0.179458	1.810895	0.151794	1.809064	0.087033
COMT	0.448983	0.021077	0.441362	0.027647	0.459967	0.019471
GAD65	1.814011	0.048398	1.706021	0.104366	1.732414	0.063425
AHD2	0.005878	0.001043	0.009950	0.002562	0.003542	0.000501
DLK1	1.524257	0.126563	1.292695	0.056949	1.361457	0.070035
Ki67	0.155954	0.011839	0.162774	0.011133	0.215717	0.020321

siRNA, small interfering RNA; *HOTAIRM1*, HOX-antisense intergenic RNA myeloid 1; *TH*, tyrosine hydroxylase; *Lmx1a*, LIM homeodomain gene 1a; *Ngn2*, neurogenin 2; *Nurr1*, nuclear receptor related 1 protein; *COMT*, Catechol-O-Methyltransferase; *GAD65*, Glutamate decarboxylase 65; AHD2, aldehyde dehydrogenase-1a1; DLK1, Delta Like Non-Canonical Notch Ligand 1.

Table S4. In vivo E17 raw PCR data (^dCT)

	**Control siRNA (n=9)**		***HOTAIRM1* siRNA (n=6)**	
	Mean	SEM	Mean	SEM
TH	0.076048	0.002810	0.059451	0.004944
lmx1a	0.063052	0.004347	0.048026	0.002282
Ngn2	0.000777	0.000054	0.000849	0.000103
Nurr1	0.331911	0.014314	0.262107	0.017453
DAT	0.059102	0.003167	0.040410	0.003987
VMAT2	0.138197	0.002727	0.114264	0.007866
MAOa	0.581047	0.012495	0.568935	0.008390
GAD65	0.890737	0.012945	0.815939	0.039680
COMT	0.240018	0.006924	0.232185	0.006513
DLK1	0.192695	0.009065	0.177225	0.007809
AHD2	0.129625	0.006915	0.093531	0.011899
Ki67	0.049776	0.003538	0.061484	0.007711

siRNA, small interfering RNA; *HOTAIRM1*, HOX-antisense intergenic RNA myeloid 1; *TH*, tyrosine hydroxylase; *Lmx1a*, LIM homeodomain gene 1a; *Ngn2*, neurogenin 2; *Nurr1*, nuclear receptor related 1 protein; *DAT*, dopamine transporter; *VMAT2*, vesicular monoamine transporter 2; *MAOa*, monoamine oxidase A; *GAD65*, Glutamate decarboxylase 65; *COMT*, Catechol-O-Methyltransferase; *DLK1*, Delta Like Non-Canonical Notch Ligand 1; *AHD2*, aldehyde dehydrogenase-1a1.

Figure S3. Representative blots for tyrosine hydroxylase protein levels

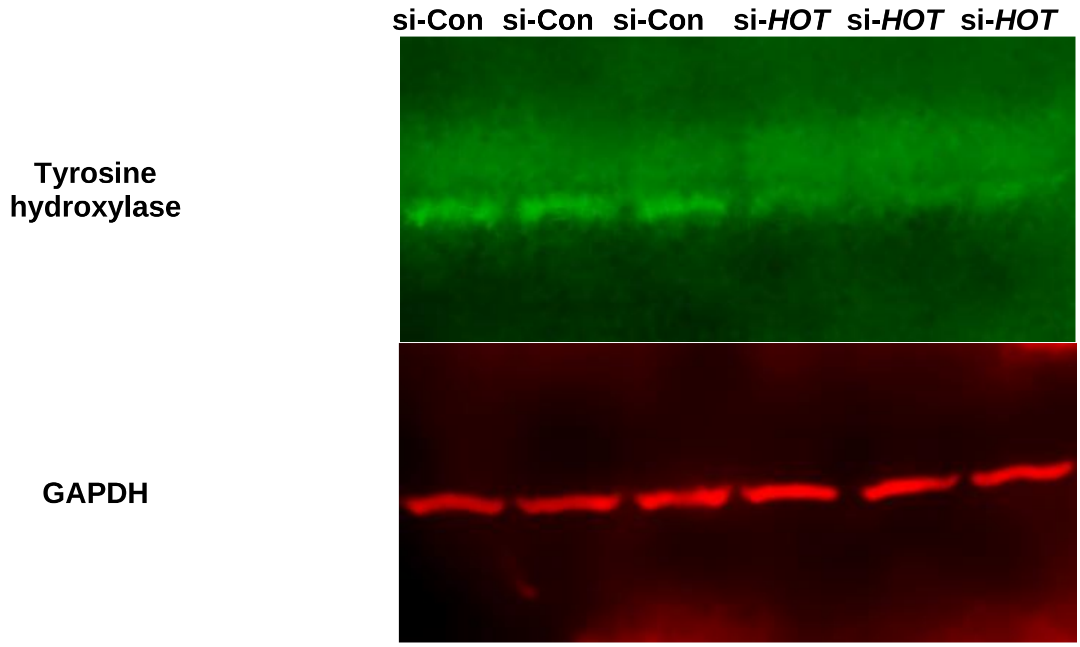

si-Con, control small interfering RNA; *si-HOT*, HOX-antisense intergenic RNA myeloid 1 targeted small interfering RNA; *TH*, tyrosine hydroxylase; *GAPDH*, Glyceraldehyde 3-phosphate dehydrogenase.

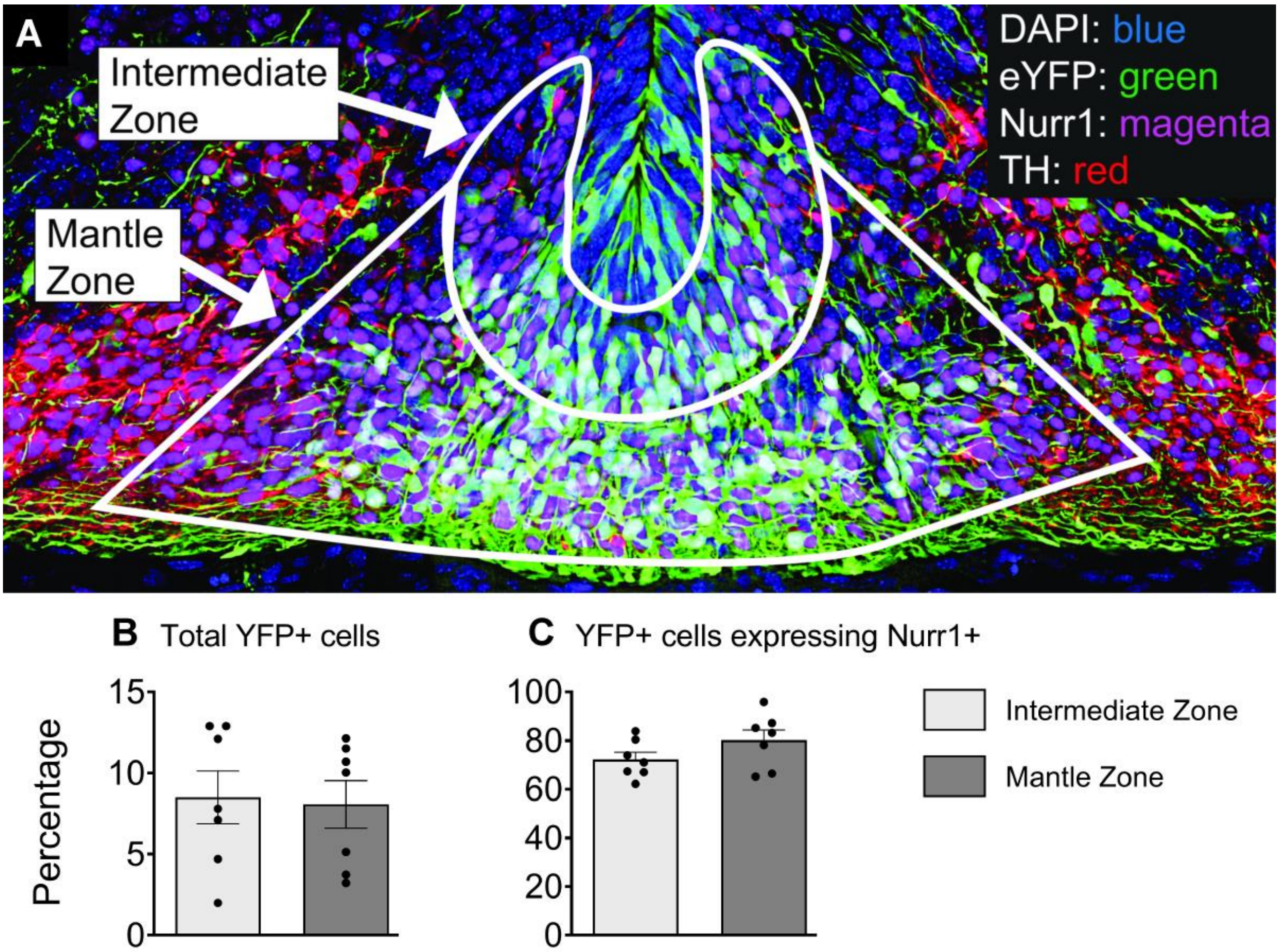

Figure S4. Proportion of cells tagged at E13 with eYFP after *in utero* electroporation at E11. The number of eYFP+ cells and Nurr1+ cells were quantified in the intermediate zone and mantle zone of E13 embryos (**A**). Levels of transfected cells were similar between subregions with approximately 8% of all cells in the ventral midbrain expressing eYFP (**B**). The majority of eYFP transfected cells were also Nurr1+ (**C**) suggesting most transfected cells were immature dopamine cells**.** *eYFP*, enhanced yellow fluorescent protein; *Nurr1*, nuclear receptor related 1 protein. Data are expressed as Mean ± SEM.

SUPPLEMENTARY REFERENCES

1. McQuin, C.; Goodman, A.; Chernyshev, V.; Kamentsky, L.; Cimini, B. A.; Karhohs, K. W.; Doan, M.; Ding, L.; Rafelski, S. M.; Thirstrup, D.; Wiegraebe, W.; Singh, S.; Becker, T.; Caicedo, J. C.; Carpenter, A. E., CellProfiler 3.0: Next-generation image processing for biology. *PLoS biology* **2018,** 16, (7), e2005970.
